# Whole-Genome and Transposed Duplication Contributes to the Expansion and Diversification of *TLC* Genes in Maize

**DOI:** 10.3390/ijms20215484

**Published:** 2019-11-04

**Authors:** Weina Si, Tianlu Hang, Mingyue Guo, Zhen Chen, Qizhi Liang, Longjiang Gu, Ting Ding

**Affiliations:** 1 National Engineering Laboratory of Crop Stress Resistance Breeding, School of Life Sciences, Anhui Agricultural University, Hefei 230036, China; Weinasi@ahau.edu.cn (W.S.); hangtianlu1995@163.com (T.H.); gmygmya@163.com (M.G.); cqc13095458229@163.com (Z.C.); liangqizhi@ahau.edu.cn (Q.L.); 2 School of Plant Protection, Anhui Agricultural University, Hefei 230036, China

**Keywords:** *TLC* genes, gene duplication, subfunctionalization, maize

## Abstract

TLC (TRAM/LAG/CRN8) proteins play important roles in ceramide metabolism and mycotoxin resistance. Herein a comparative genomics analysis of TLCs was performed in 31 plant and 3 species from other kingdoms, with an emphasis mainly on maize. TLCs were conserved across kingdoms and expanded in angiosperms, largely due to whole-genome/segmental duplication (WGD/SD) under purifying selection. Phylogeny reconstruction by maximum-likelihood method uncovered five TLC clades, subsequently named as TRAM/LAG, CLN8, PS-TLC, TM136 and TLCD clades. Each clade of TLCs shared specific transmembrane regions and motif composition. Divisions of conserved motifs to subunits may have occurred in TM136-type TLCs. Focusing on maize, five WGD and two DNA-mediated transposed duplication (TD) pairs were discovered, accounting for 61.11% *ZmTLCs*. Combined with further expression analysis, significant divergence was found in expression patterns between most maize WGD pairs, indicating subfunctionalization or/and neofunctionalization. Moreover, *ZmTLC5*, a deduced parental copy in a TD pair, was highly induced under FB_1_ and fungus pathogen injection and exhibited potential capacity to respond to environmental stimuli. Additionally, population genetics analysis showed that *ZmTLC10* in the CLN8-clade may have experienced significant positive selection and differentiated between wild and inbred maize populations. Overall, our results help to decipher the evolutionary history of *TLCs* in maize and plants, facilitating further functional analysis of them.

## 1. Introduction

Plants are sessile and vulnerable to various biotic stresses, including bacteria, fungi, oomycetes, and nematodes [1]. In the long-term evolution process, plants have developed multiple immune defense routes to against pathogen invasion [2]. Disease resistance (*R*) genes have an irreplaceable role in the immune defense system, responding to pathogen virulence effectors and conforming to “gene-to-gene” hypotheses [3]. Among the various *R*-genes, *TRAM*/*LAG*/*CLN8* (*TLC*) genes cause less concern. They are different from most *R*-genes and render plant resistance to pathogenic toxins, including FB_1_ (fumonisins B_1_) and AAL (*Alternaria alternata* f. sp. *lycopersici*) toxins, generally produced by *Fusarium* and *Alternaria* genus fungus, respectively. These toxins not only threaten crop quality and storage, but also harm animal and human health.

*TLC* genes are mainly comprised of *LAG* (longevity assurance gene), *TRAM* (translocating chai-associating membrane protein) and *CLN8* (Neuronal Ceroid Lipofuscinosis 8) members [4]. They have been systemically investigated mainly in human, animal, and yeast encoding membrane-associated proteins [5]. It is summarized that these three types of TLC members are ER (endoplasmic reticula)-resident TM (transmembrane) proteins and ubiquitous in the eukaryotic kingdom. They act as synthases and activate the synthesis of ceramide-like moieties and/or sphingolipids, and translocate proteins in the ER [4]. It was the LAG-type *TLC* genes that were first reported to act as ceramide synthases and exhibit resistance to FB_1_ and AAL toxins [6]. Besides *LAG* genes, *CLN8* and *TRAM* genes are also notable, they are important in stress response or apoptosis [7,8,9].

Ceremide synthesis in sphingolipid metabolism is also critically important in plants. The sphingolipid metabolism pathway, including ceremide synthesis, plays an important role in biotic and abiotic stress responses, triggering programmed cell death in plants [7,10,11]. In-depth investigation of *TLC* genes would be beneficial not only for decomposition of the sphingolipid metabolism, but also for disease therapy or food security. However, compared with animals and yeast, research about *TLC* genes in plants have been rarely reported. Available studies are mainly about LAG-type *TLC* genes in limited plant species. The first reported *LAG* gene in plants is *Alternaria stem canker locus*-*1* (*asc1*) in *Solanum lycopersicum*, which endowed plants with resistance to FB_1_ and AAL toxins. Three *LAG* homologs in *Arabidopsis thaliana*, *AtLOH1*, -*2*, and -*3*, encode three different isoform ceramide synthases with distinct substrate specificities. Ceramide synthases are central in sphingolipid metabolism and catalyze the formation of ceramides from sphingoid long-chain base (LCB) and fatty acyl-CoA substrates [12]. *AtLOH1* and *AtLOH3* catalyze reactions with very-long-chain fatty acyl-CoA and trihydroxy long-chain base (LCB) substrates, while *AtLOH2* applied palmitoyl-CoA and trihydroxy long-chain base (LCB) substrates. Furthermore, *AtLOH1* ceramide synthase is strongly inhibited by FB_1_, while *AtLOH2-* and *AtLOH3*-overexpressing plants are tolerant to FB_1_ toxicity [11]. As one of the most important cereal crops worldwide [13], maize (*Zea mays*) suffers increasingly worse fungal diseases, causing mycotoxins to be an important threat to maize production and quality [14]. However, few reports about mycotoxin *R*-genes, *TLC* genes, have been published for maize. The completion of genome sequences of maize and many other sequences provide us valuable genetic data to decipher the evolutionary fate and characteristic of *TLC* genes in maize and plant kingdoms, which could be a stepping stone for in-depth functionally characterization of *TLC* genes [15]. 

In this study, TLC proteins were genome-wide identified from the maize genome. Their duplication modes, collinear relationship with monocot and dicot species, TM regions and conserved motif organization, gene exon/intron structure, population genetics, tissue-specific expression profiles, and expression patterns under toxin and pathogen injection were systemically investigated. Specifically, *TLC* genes from 30 other plant species and 3 outgroup species were also employed in this study to better reconstruct phylogeny and trace the evolutionary history of plant and maize TLC proteins. The results presented would not only uncover the evolutionary fate of *TLCs* in maize and plants, but also provide theoretical support for the future functional analysis of *ZmTLCs*.

## 2. Results

### 2.1. Identification of TLC Genes from Maize and 30 Other Plant Species

**Figure 1:**
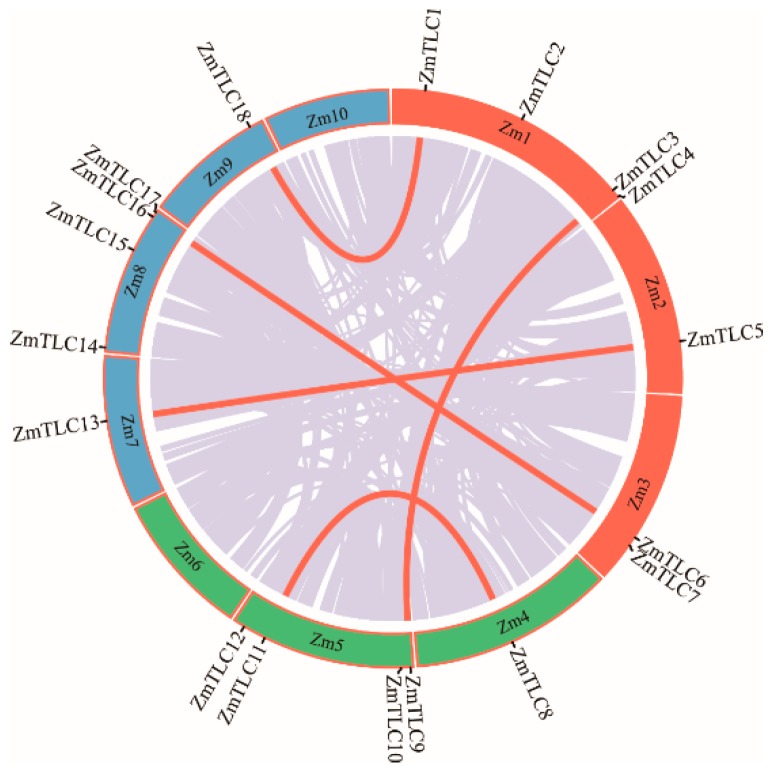
Chromosomal distribution of maize *TLC* genes. Genome-wide collinear genes in maize are linked with grey lines and collinear *TLC* genes are marked with red lines.

*TLC* homologs from maize were characterized by a genome-wide Pfam homology search and 18 *ZmTLCs*, encoding proteins harboring the TRAM_LAG1_CLN8 domain, were detected. These 18 *ZmTLCs* homologs were distributed unevenly on eight of ten maize chromosomes (Figure 1). Four *ZmTLC* genes reside in chromosome 1, 5, and 8, respectively. Two are located in chromosome 3, followed by only one in chromosome 2, 4, 7, and 9. These 18 *ZmTLC* genes were further named as *TLC1* to *TLC18* according to their chromosomal distribution (Table 1). In addition, the length of ZmTLC proteins were relatively conserved, ranging from 189 aa to 331 aa, with an average length of 279 aa. The iso-electricpoint (pI) and molecular weight (Ww) of 18 ZmTLC proteins were also calculated and listed in Table 1.

**Figure 2:**
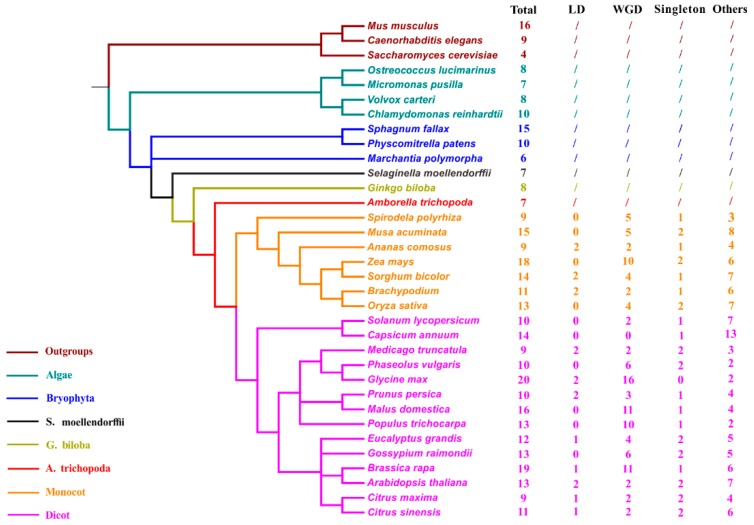
Taxonomic tree of 31 surveyed plant species and 3 outgroup species. The total number of genome-wide identified *TLCs* in each species and number of *TLCs* involved in different duplication-modes were also listed. Species from different taxonomies and/or species were marked with different colors. “Total” represents total TLC protein numbers in each species; “LD” represents local duplication, including tandem and proximal duplication; “WGD” represents Whole-genome/Segmental duplication; “/” means duplication mode was not estimated.

*TLC* homologs were also explored from 30 other representative plant genomes to better confirm the evolutionary status of *TLC* genes (Figure 2; Table S1). These 30 plant species cover 22 plant families, ranging from aquatic alga to higher angiosperms. *TLC* genes of *Saccharomyces cerevisiae* from the fungus kingdom and *Caenorhabditis elegans* and *Mus musculus* from the animal kingdom, were simultaneously detected and utilized as outgroups for further analysis. Results showed that *TLC* genes are conserved across all surveyed lineages. The four algae genomes harbored an average of eight *TLC* genes, with small ranges from 7 to 10. In the basal plant genomes, including three moss species, *Physcomitrella patens*, *Sphagnum fallax*, and *Marchantia polymorpha*; one fern species, *Selaginella moellendorffii*; and one gymnosperm, *Ginkgo biloba*, 6 to 15 *TLC* genes were identified. In higher green lineages, including 21 angiosperm species, there were variable *TLC* copy numbers from 6 to 20. Among all these surveyed species, *Glycine max* had the most *TLC* genes, yielding as many as 20, while *Amborella trichopoda*, the common ancestor of dicots and monocots, harbored the lowest *TLC* copy number. In *C. elegans*, *S. cerevisiae* and *M. musculus*, 9, 4, and 16 *TLC* genes were identified, respectively. Correlation between the number of *TLC* genes and genome size, chromosome number and genomic-wide protein-encoding gene number, were also assessed. Results demonstrated that the number of *TLC* genes was positively correlated with the genome-wide protein-encoding gene number for each genome (Figure S1) (r^2^ = 0.67, *p* < 0.01; Pearson’s product–moment correlation). 

### 2.2. Identification of Modes of Gene Duplication in Plant and Maize TLC Homologs

In surveyed plant species, the *TLC* gene family showed different degrees of expansion, especially in angiosperms. Gene duplication, generally resulting in multiple duplication modes including WGD/SD, local duplication (LD, including tandem and proximal duplication) and transposed duplication (TD), contributes mainly to gene family expansion in angiosperms [16,17]. In particular, WGD/SD and LD are considered as the two main causes of gene family expansion. Thus, to decipher mechanism underlying *TLC* gene expansion in plant species, Blastp and MCScanX were applied to first detect WGD/SD and LD events involved in the *TLCs* of angiosperms (Figure 2) [18,19]. In surveyed angiosperms, 7.9% *TLC* genes were singletons and others were involved in a kind of duplication mechanism. WGD/SD pairs made up 40.4% (107 out of 265) of *TLC* genes, while only 6.8% of *TLC* genes were LD gene pairs. LD modes could be detected from only 10 of the 22 angiosperm species.

**Figure 3:**
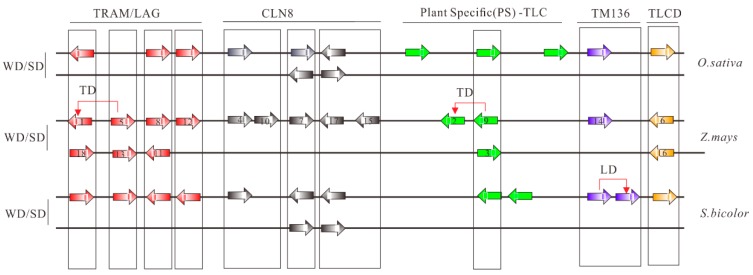
Collinear analysis of *TLC* genes between maize and other species, including *S. bicolor* and *O. sativa*. TLC proteins are shown as arrows. Arrows towards the right direction means the gene was in the forward strand orientation, while arrows in the left direction means the gene was in the reverse strand orientation. Arrows with different colors represent different TLC types. Collinear communities are marked with black boxes. Results of collinear analysis between maize and *Arabidopsis* were not shown due to no collinear communities being found.

Concentrating on maize, no LD events were identified and 10 of 18 (55.56%) *TLC* homologs were WGD duplicated pairs (Figure 3). Hence, in the remaining eight maize *TLC* genes, potential TD events were also characterized. To obtain accurate results, four genomes with a close genetic relationship with maize, including *Oryza sativa*, *Setaria italica*, *Brachypodium distachyon*, and *Sorghum bicolor*, were employed as outgroups. It is presumed that *ZmTLC9* and *ZmTLC5* are parentally copied, generating *ZmTLC2* and *ZmTLC1* by transposed duplication, respectively (Figure 3). 

### 2.3. Calculation of Evolutionary Parameters of TLC Paralogs

Estimation of the evolutionary ratio of *TLC* duplicated paralogs would be beneficial for understanding dynamics driving *TLC* gene family evolution in plant kingdoms. Ka/Ks ratio is one of the most important molecular evolutionary parameters. Generally speaking, Ka/Ks > 1 indicates evolution under positive selection pressure, Ka/Ks < 1 is said to be conserved and subjected to purifying selection, and Ka/Ks = 1 indicates neutral selection. In the present study, measurement of Ka/Ks was performed to trace the evolutionary force responsible for WGD and LD *TLC* duplicated pairs. The majority of those WGD and LD duplicated pairs were under “purifying selection”, showing few or no selection constraints. Only one LD type gene pair, *Eucgr.G01439* and *Eucgr.G01442*, had Ka/Ks bigger than 1 (Table S4).

**Figure 4:**
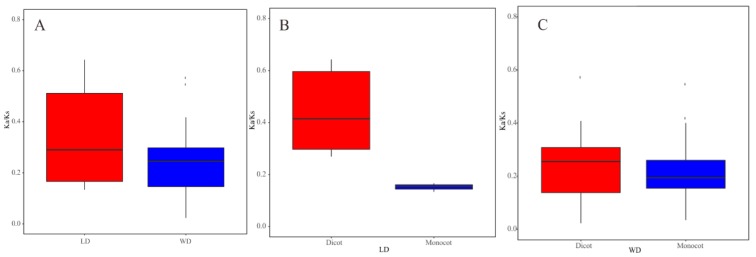
Boxplots of non-synonymous (Ka) to synonymous (Ks) ratio of WGD and LD duplicated pairs. (**A**) Ka/Ks values of WGD and LD *TLC* genes pairs in all angiosperm plants. (**B**) Ka/Ks values of LD *TLC* gene pairs in dicot and monocot plants. (**C**) Ka/Ks values of WGD *TLC* gene pairs in dicot and monocot plants.

According to our data, WGD and LD duplicated pairs had different evolutionary rates (Figure 4). Comparing the Ka/Ks values of WGD with those of LD duplicated pairs in all angiosperms, the average Ka/Ks value of WGD duplicated pairs (0.23) was less than that of LD duplicated pairs (0.42). Furthermore, LD duplicated pairs in monocots had a smaller average Ka/Ks value than those in dicot plants. This is consistent with the result that WGD pairs had a higher average value in dicots than that in monocots. These results demonstrated that there is a distinct molecular evolutionary ratio between LD and WGD *TLC* gene pairs, as well as *TLC* gene pairs between monocot and dicot plants. Additionally, the two TD pairs in maize also underwent purifying selection, with Ka/Ks lower than 1 (Table S4).

### 2.4. Contruction of a Maximum Likelihood (ML) Phylogenetic Tree with TLCs from All Surveyed Species

**Figure 5:**
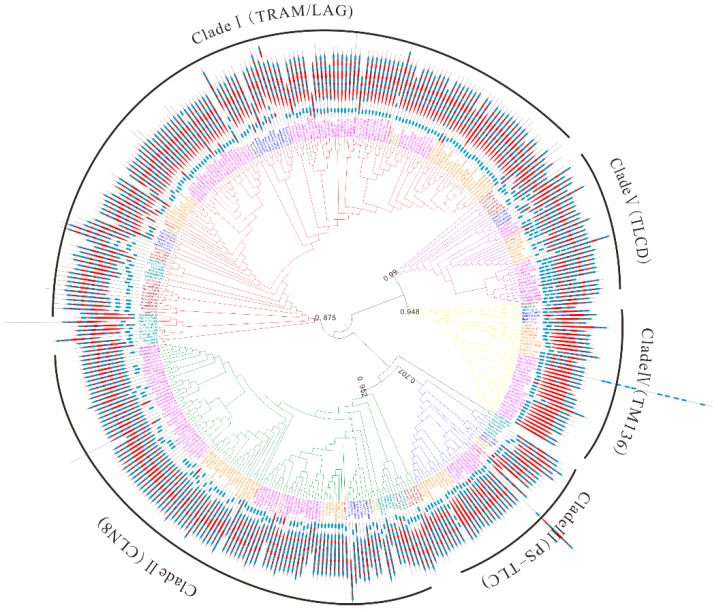
The maximum likelihood (ML) phylogenetic tree built by TLC proteins from 31 plant species and 3 outgroup species. Abbreviation of species names in Table S1 were used as prefixes in the protein names to denote the species they belong to. Additionally, TLC members belonging to different lineages or/and species were marked in the same colors as those in Figure 2. The phylogenetic tree could be categorized into five clades and branches of different clades were marked with different colors. Red, green, blue, yellow, and purple branches represent Clade I to V, respectively. Transmembrane (TM) region topology across TLC proteins is also shown. Blue squares represent TM regions and red diamonds represent TRAM/LAG/CLN8 domains.

**Figure 6:**
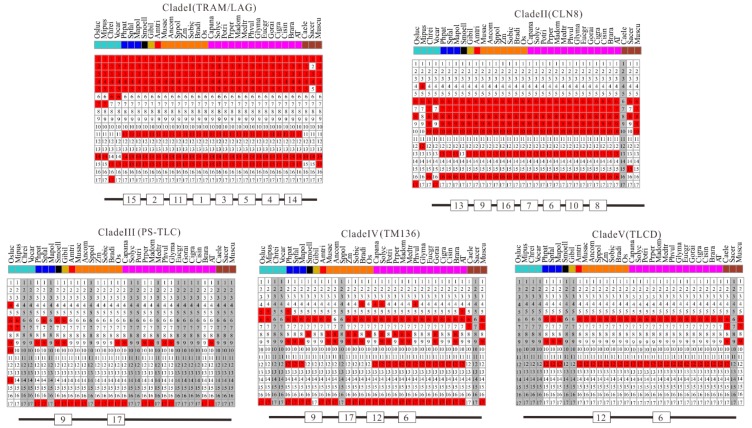
Conserved motif composition of TLC proteins in five clades. Schematic representations of the 17 conserved protein motifs in TLC proteins from 31 different plant species and 3 outgroup species in five clades. Numbers in squares are motif accession numbers. The red and white indicate the presence of motifs or not. Gray means no sequences from the species could be found in corresponding clades.

To better trace the evolutionary history of *TLC* genes in maize and plant kingdoms, a maximum likelihood (ML) phylogenetic tree was obtained with 348 TLC proteins from 31 plant species and 29 TLC proteins from 3 outgroup species (See Materials and Methods). According to the topology of the phylogenetic tree and taxonomy of surveyed species in Figure 2 (Figure 5 and Figure S2), the phylogenetic tree could be categorized to five clades, subsequently named as Clade I–V. TLC proteins from different species were unevenly clustered within different clades (Figure 5 and Figure 6). There were 155, 113, 34, 38, and 37 TLCs in the five clades. Clade I harbored the most TLC members, while Clade III harbored the least. In addition, Clade I comprised TLC proteins from all 34 genomes, including the three outgroup species. Clade II harbored TLC proteins from most surveyed genomes, except for one outgroup specie, *C. elegans*. In the other three clades, TLC proteins were absent in several species, or/and even lineages. For example, TLCs from all three outgroup species were absent in Clade III. Thus, this clade was further suggested to be a plant-specific (PS) TLC clade. In Clade IV, TLC proteins could be found in almost all surveyed lineages, while absent in several species. Finally, neither TLC homologs from the four aquatic alga nor the fungus *S. cerevisiae* were present in Clade V. According to the annotation of previously reported TLC proteins in *M. musculus* and their corresponding distribution [20], TRAM- and LAG- type TLCs were clustered within Clade I, while CLN8-type TLCs were in Clade II. TLCs in Clade IV were closely related to TM136, and TLCD5 was closely related to proteins in Clade V. Consequently, proteins in Clade I to V were annotated as TRAM/LAG-, CLN8-, PS-TLC-, TM136-, and TLCD- type TLC proteins in further analysis.

### 2.5. TM Regions and Conserved Motif Organization in TLC Proteins within Different Clades

It is reported that TLCs have at least five TM α helices [4]. Our research first inspected TM region topology in TLC proteins with SMART (Figure 5). The above results highlight that TLC proteins in plant kingdoms could be subdivided to five well-supported clades. Results demonstrated that TLC proteins in different clades shared distinct, common, TM region composition. TLC proteins in Clade I consisted of five TM regions, with four TM regions forming the TLC domain and one in the N-terminal to the TLC domain. In Clade II, the majority of the TLC proteins harbored six predicted TM regions, with five TM regions forming the TLC domain and one N-terminal to the TLC domain. In Clade III, the number of predicted TM regions varied, with two to six TM regions constituting the TLC domain and one adjacent to the N-terminal of TLC domain. In Clade IV, the majority of the TLC domain consisted of two TM regions, with one TM region adjacent to its N-terminal. In Clade V, proteins had five predicted TM regions in the TLC domain, with two TM regions adjacent to the N-terminal of the TLC domain.

**Figure 7:**
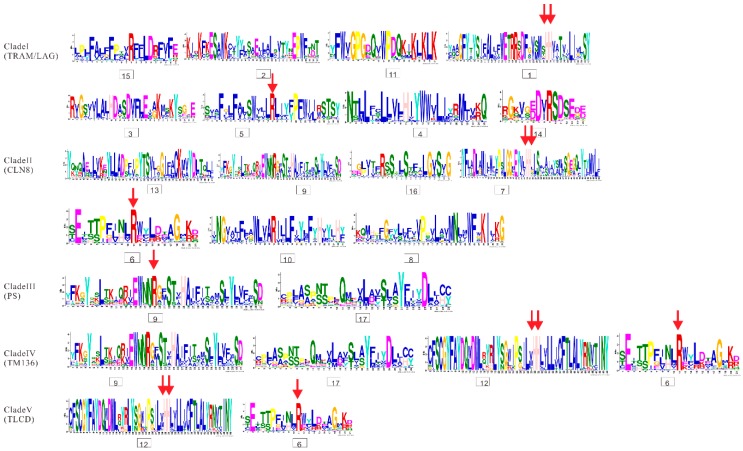
Sequence logos for conserved motifs in proteins of five clades. The results were produced by the Multiple Em for Motif Elicitation (MEME) program. The conserved Arg (R) residue and two consecutive histidine residues are marked with red arrows.

Additionally, all the protein sequences of TLCs were submitted to the MEME website simultaneously to decipher their conserved motif composition. These TLC sequences showed large motif composition divergence. Only when the motif number was set to as many as 17 could significant conservation be detected within most surveyed TLC sequences (Figure 6). Though high motif composition divergence was in TLC proteins across different clades, proteins within the same clades shared very similar conserved motif composition. Motif 1, 2, 3, 4, 5, 11, 14, and 15 were conserved in more than half of the TLC proteins in Clade I. Clade II had conserved motifs including motif 13, 9, 16, 7, 6, 10, and 8. Motif 9 and 17 were conserved in Clade III. Clade IV had conserved motif 9, 17, 12 and 6, while Clade V had conserved motif 12 and 6. From detailed analysis of the residues in motifs, the absolutely conserved Arg (R) residue mentioned in previous reports could be found in all five of the clades (Figure 7). However, the two consecutive histidine residues (“HH”) in previous reports, which may be related to catalysis or substrate binding, could only be detected in four of the five clades, except for Clade III, which only harbored plant TLCs [12].

### 2.6. Phylogeny Reconstruction and Intron/Exon Structure Analysis of Maize TLCs

**Figure 8:**
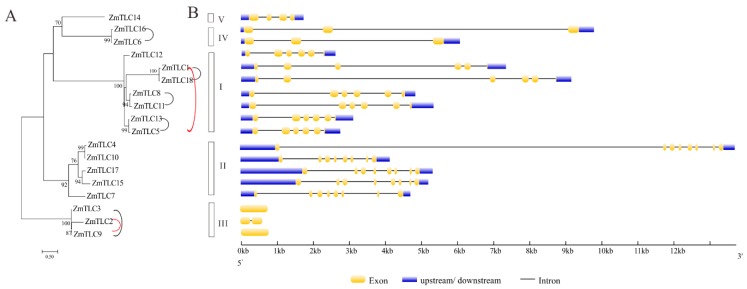
The ML phylogenetic tree built by ZmTLC proteins (**A**) and their exon/intron composition (**B**). This ML phylogenetic tree could also be divided into five clades, in which ZmTLCs showed almost the same topological relationship as those in the ML tree in Figure 5. WGD TLC gene pairs are linked by black lines; TD gene pairs are linked by red lines.

Phylogeny reconstruction with TLC proteins from 22 representative plant families would be beneficial for the classification and evolutionary history of maize TLC proteins. In the above ML phylogenetic tree based on 34 species, Clade I, also named as TRAM/LAG Clade, harbored as many as seven maize TLC proteins, including ZmTLC1, -5, -8, -11, -12, -13, and -18. In Clade II, there were five maize TLC proteins, including ZmTLC4, -7, -10, -15, and -17. In addition, ZmTLC2, -3, and -9 clustered within Clade III, and ZmTLC14 clustered within Clade IV. ZmTLC6 and -16, could be identified in Clade V. These ZmTLCs within the same clade had very similar TM and motif composition features, consistent with the common properties we summarized from the above 34 species phylogenetic tree (Figure 5 and Figure 6). To confirm the reliability of the ML tree, we also constructed another phylogenetic tree, with only TLC proteins from maize (Figure 8A). As expected, maize TLC proteins displayed almost the same topological relationship between the two trees. The new tree could also be subdivided into five clades, in which ZmTLCs were distributed the same as the above ML phylogenetic tree. A similar intron/exon distribution pattern can be observed for ZmTLC proteins in each clade (Figure 8B). For example, genes in Clade I harbored five introns; *TLC* genes in Clade II had seven introns; *TLC* genes in Clade III have no or one intron; *TLC* genes in Clade IV contained two introns; *TLC* genes in Clade V contained three introns.

### 2.7. Collinear Analysis of TLCs Across Maize and Three Other Species

Phylogenetic trees preliminarily revealed the homologous relationship between TLCs in maize and other species. Collinear or/and synteny analysis enabled us to better illustrate the origins of ZmTLCs [21] (Figure 3). Applied with two representative grass genomes, including *O. sativa* and *S. bicolor,* and one dicot genome, *Arabidopsis*, a synteny-based method was employed to explore collinearity and orthology between maize and other grass and dicot lineages. Surprisingly, no synteny communities of TLCs could be found between maize and *Arabidopsis*, showing their distant and obscurely orthologous relationship. However, TLC proteins were largely collinear across the grass lineages. Nine collinearity communities were discovered among maize and two other grass species, covering 77.78% (14 out of 18) ZmTLCs. One collinearity community was found only between maize and *S. bicolor*. These syntenic orthologs were likely to be descended from a common ancestral gene before speciation.

### 2.8. Population Genetics Analysis of ZmTLC in Inbred and Wild Maize

More and more re-sequencing data provides us with an unprecedented chance to perform molecular population genetics analysis, which could apply within- and between-population DNA sequence variation to trace evolutionary dynamics in maize populations. Re-sequencing data for 30 inbred and 22 wild maize were applied in the present study [22]. Nucleotide diversity (π) was firstly calculated using DnaSP to estimate the population mutation rate (Table 2). π presents the average number of nucleotide differences between two sequences within one population. Results showed the level of π varied across different *TLC* genes in both wild and inbred maize populations. In inbred maize, π ranged from 0 at *ZmTLC1* to 0.08 at *ZmTLC7*, while π in wild maize ranged from 0 at *ZmTLC1* to 0.7 at *ZmTLC7* and *-4*. The average π (0.003) of all TLC loci in inbred maize population is lower than that (0.004) in the wild maize population. In five loci, *ZmTLC4*, *-6*, *-10*, *-14*, and *-17*, π within the inbred maize population is higher than that in the wild maize population. In seven loci, *ZmTLC1*, *-5*, *-8*, *-12*, *-13*, *-16*, and *-18*, π within the inbred maize population is equal to that in the wild maize population. Apparently, *ZmTLC1* showed no single gene polymorphism (SNP) within both the inbred and wild maize population, indicating the genetic conservation in maize. At *ZmTLC7*, π is the highest in both inbred and wild maize, and it is the only locus at which π is higher in the inbred population than in the wild maize population. 

One neutrality test, the Tajima’D test, was performed to detect selection pressure under individual loci [23] (Table 2). The Tajima’D statistic is expected to be 0 according to the standard neutral equilibrium model. Positive D values imply an excess of intermediate frequency polymorphisms, while negative Tajima’D values speculate rare polymorphism. In the present study, mean Tajima’D values were 0.11 in the inbred maize population and −0.67 in the wild maize population. This is consistent with a previous study which found that SNP frequency in wild maize is skewed from neutral expectations, implying population expansion or subdivision [24]. As to individual loci, only one CLN8-type locus, *ZmTLC10*, showed a statistically significant (*p* < 0.05) Tajima’D value (−1.73). Moreover, the Tajima’D value of *ZmTLC10* in inbred maize is the lowest among all surveyed loci, indicating positive selection or linkage to a swept gene.

Finally, an important parameter, Fst, the measurement of the genetic differentiation between inbred and wild maize populations, was also calculated (Table 2). Generally, the closer Fst is to 0, it implies a lower degree of divergence between two surveyed populations. The closer Fst is to 1, it indicates a higher degree of differentiation between populations. The average value of Fst between inbred and wild maize was 0.12 for *ZmTLC* genes, showing overall slight differentiation. Four loci showed statistically significant Fst values between these two maize populations, including *ZmTLC4*, *-6*, *-8*, and *-10*, indicating a high level of genetic differentiation at these four loci between two maize populations.

### 2.9. Expression Profiles of ZmTLC Genes in Different Tissues

**Figure 9:**
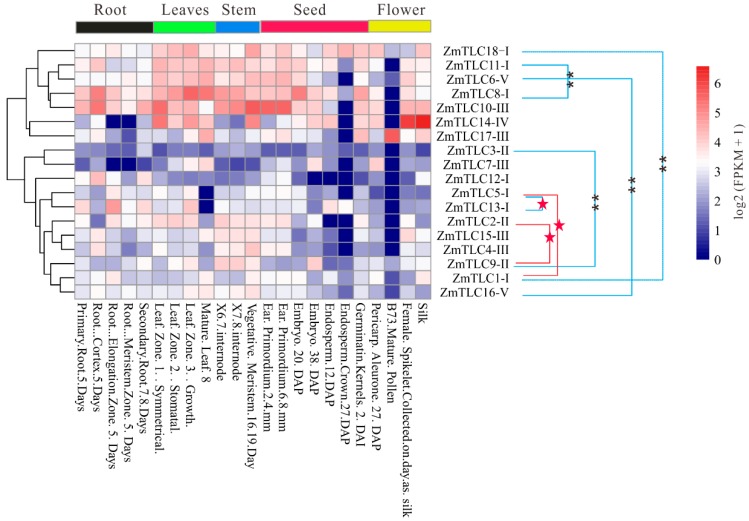
Tissue-specific expression profiles of *ZmTLC* genes. Expression pattern divergence between WD/SD gene pairs (linked by blue lines) and TD gene pairs (linked by red lines) were estimated by one-way ANOVA test. ** represents highly significant divergence (*p* < 0.01) and red stars represent no significant divergence (*p* > 0.05).

In the present study, gene expression patterns of *ZmTLCs* were analyzed across 14 organs/tissues involved in 23 diverse developmental stages, to yield their high-resolution spatial expression profiles in the life cycles of maize and promote further functional analysis (Figure 9). *ZmTLC* genes displayed diverse tissue-specific expression patterns. *ZmTLC1, -9*, *-16*, and *-18* were expressed in all tested developmental phases. *ZmTLC8* and *-10* showed a relatively high level of expression in almost all tested developmental phases (FPKM ≥ 10), while *ZmTLC1*, *-3*, *-5*, and *-16* exhibited a relatively low level of expression in most surveyed developmental phases. *ZmTLC15*, *-9*, *-17*, *-2*, *-4*, and *-10* were hardly expressed in endosperm. The expression level of most *ZmTLCs* in mature pollen was equal to zero, whereas *ZmTLC17* was highly expressed in mature pollen. On the other hand, most *ZmTLC* genes within the same clade exhibited distinctive k means clustering of expression values, exhibiting expression pattern or functional diversity. Intriguingly, four of the five WGD duplicated pairs, *ZmTLC1* and *-18*, *-8* and *-11*, *-6* and *-16*, *-3* and *-9*, revealed significant diverse tissue-specific expression patterns (one-way ANOVA test, *p* < 0.01) [25] (Figure 9). Whereas the two transposed duplication pairs, *ZmTLC1* and *-5*, *-2* and *-9*, manifested expression profiles across tissues with no significant diversity (one-way ANOVA test, *p* > 0.05).

### 2.10. The Relative Expression Levels of ZmTLC Genes in Response to FB_1_ Toxins and Pathogens

**Figure 10:**
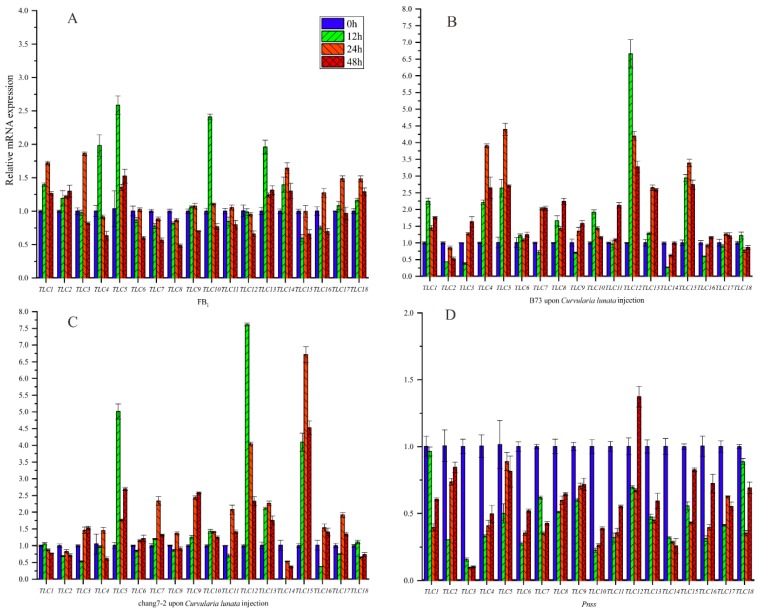
Relative expression level of *ZmTLC* genes under different treatments at four time points. (**A**) Fumonisins B_1_ (FB_1_) injection into B73 inbred; (**B**) *Curvularia lunata* injection into B73; (**C**) *Curvularia lunata* injection into chang7-2; (**D**) Pnss injection into one sweetcorn.

Previous experimental evidence proved that *TLC* genes played important roles in response to mycotoxins, including FB_1_. Maize is severely threatened by kinds of mycotoxins. Hence, we first evaluated the expression levels of *ZmTLCs* under FB_1_ irrigation in maize inbred B73 at four time points, to deduce the potential role of *ZmTLCs* in response to FB_1_ (Figure 10). Two of the eighteen *ZmTLCs* were up-regulated more than two-fold at one or several time points, including *ZmTLC5*, *-10*, and *-13*. As kinds of fungus could produce toxins [26], we also assessed the relative expression levels of *ZmTLC* genes after the infection of one fungus pathogen, *Curvularia lunata,* to B73 and another important inbred, chang7-2 (Figure 10). Additionally, one bacterial pathogen, *Pantoea stewartii* ssp. *stewartii* (Pnss) was used as a comparison and the expression levels of *ZmTLCs* were also detected in sweetcorn, T1, under the injection of Pnss. Consequently, the expression levels of *ZmTLC -5*, *-12*, *-13*, and *-15* were highly induced under the injection of *Curvularia lunata* in two samples. On the contrary, all the *ZmTLC* genes were down-regulated under the induction of Pnss, which could cause bacterial blight disease in maize. Overall, *ZmTLC5* was highly induced, both under the irrigation of FB_1_ or upon the infection of *Curvularia lunata*, implying its potential role in response to mycotoxins.

## 3. Discussion

TLCs have been previously identified as TM proteins, mainly composed of TRAM, LAG, and CLN8 members. They are believed to participate in ceramide/lipid synthesis [5], functioning as ceramide synthase. Ceramide is an important skeleton of sphingolipids, which form a majority of membrane structure, regulate signaling pathways, and play an important role in response to biotic and abiotic stress [7]. Conforming to their functionally irreplaceability, *TLC* genes were evolutionarily conserved and have ancient origins across biology kingdoms, as *TLC* genes could be identified in all our surveyed species (Figure 2). On the other hand, in our results, *TLC* paralogs were of dramatic evolutionary diversity, and phylogeny reconstruction with TLC proteins from all surveyed species revealed that TLC proteins could be divided into five obvious clades, indicating evolutionary diversity. TRAM and LAG members clustered closely in Clade I, coinciding with the reports that *TRAM* and *LAG* genes shared high sequence similarity [9]. Additionally, previously identified *Asc-1* from *S. lycopersicum* and ceramide synthase *AtLOH1* (At3g25540), *AtLOH2* (At1G13580) and *AtLOH3* (AT3G19260) from *Arabidopsis* were also clustered within Clade I [11,27]. Four of the five clades, TRAM/LAG, CLN8, TM136, and TLCD type *TLC* genes, harbored TLC proteins from at least one species in the outgroups, implying their ancient origins and evolutionary conservation across biology kingdoms. The PS-TLC (Clade III) clade only had TLC members from plant species, implying that these TLC proteins were either lost in other biology kingdoms or recently evolved after the differentiation of plants with other biology, which may be involved in plant specialized metabolism [28].

Conserved motifs and domains shared by homologous genes were generally believed to be important functional elements or/and regulatory molecules [29]. TLC proteins showed TM regions and motif composition divergence among members in different clades, indicating functional diversity. Previously reported conserved consecutive “HH” residues could be identified in TRAM/LAG, CLN8, TM136, and TLCD type TLC proteins, which contained yeast or animal homology [4], while no such conserved consecutive “HH” was shared by PS-TLC proteins, implying their functional specialization. Interestingly, our results suggested that PS-TLC proteins, as well as TLCD TLC proteins, were likely to originate from divisions of conserved motifs in TM136-type proteins (Figure 6). In the duplication-degeneration-complementation (DDC) model, a division of regulatory modules to subunits would generally result in subfunctionalization [30]. Our results conformed to this hypothesis, indicating the subfunctionalization consequence. It is commonly believed that subfunctionalization is the primary mechanism underlying the substrate specialization processes [31]. In the enzyme evolution, the new copy and the old would have multiple distinct modes of activity control or substrate specificity, and finally specialize in either function after subfunctionalization [32]. Furthermore, subfunctionalization has also already been detected between two TRAM/LAG-type *TLC* genes, *AtLOH1* and *AtLOH3* [11]. Compared with TRAM/LAG and CLN8 TLC proteins, rare experimental and functional characterizations could be found for TLC proteins in the PS-TLC, TLCD and TM136 clades. These outcomes would provide predication in further in-depth analysis for these three types of TLC proteins. 

The aforementioned analysis in plant kingdoms applied 31 plant and 3 outgroup species made sense to more easily classify and trace the evolutionary fate of the 18 TLC proteins in maize, which we emphasized in the present study. Maize *TLC* members shared common features across plant species, summarized based on the 34 species phylogenetic tree, no matter their topological relationships or TM regions and motif composition among or within clades. On the other hand, in our results, copy number variation and expansion occurred in *TLCs* of angiosperms. Gene duplication contributes to gene family variation and expansion in angiosperms, enabling raw genetic materials to be increased and followed by functionalization consequences, including redundancy, subfunctionalization, and neofunctionalization [31]. Our outcomes uncovered that the majority of *TLC* genes in angiosperms (40.5%) originated from WGD/SD events. Focusing on maize, 10 of 17 *ZmTLC* genes are WGD/SD duplicated gene pairs, while no LD duplicated *ZmTLC* genes were detected. Intriguingly, four of the five WGD *ZmTLC* pairs belonged to the TRAM/LAG-type. Three of the four TRAM/LAG-type WGD/SD *ZmTLC* pairs displayed significant distinct expression patterns (Figure 9), strongly indicating subfunctionalization or/and neofunctionalization, but not functional redundancy. 

TD events are another important duplication mode and were found to frequently occur in gene families such as *NBS-LRR* and *MADS*-box, which are involved in environmental adaption and disease resistance [16,33]. Adopting four evolutionarily close species, two transposed duplicated pairs were detected in maize, including *ZmTLC5* and *-1*, *ZmTLC9* and *-2*. Herein, results indicated *ZmTLC5* was an ancient parental copy and created *ZmTLC1* by TD events. The intronless *ZmTLC9* gene was more likely to be an evolutionarily ancient retrocopy, and itself may be generated by an ancient multiexon parental gene and subsequently created *ZmTLC3* by WGD and *ZmTLC2* by TD (Figure 3). Considering the presence of intron sequences in both *ZmTLC2* and *ZmTLC1* genes, they are most likely to be generated by DNA-based TD or transposon-mediated duplication [34]. These two transposed duplicated pairs shared significant similar expression levels in different tissues, indicating their distinct functional consequences compared with those WGD/SD gene pairs. Furthermore, it is fascinating that *ZmTLC5* presented potential capacity in responding to environmental stimuli in our analysis. The expression level of *ZmTLC5,* which belonged to Clade I, was highly induced either upon infection by a fungus pathogen or fumonisins B_1_. Additionally, another transposed pair, *ZmTLC9* and *-2*, belonged to plant-specific clades, which are more likely beneficial for plant-specific evolution. These results suggested that transposed duplication plays an important role in maize the evolution of TLC proteins in response to environmental stimuli or adaption. Finally, population genetic analysis revealed that a CLN8-type *TLC* locus, *ZmTLC10*, showed significant differentiation between wild and inbred maize populations and underwent significant positive/adaptive selection pressure in the inbred maize population, indicating its role in domestication. Overall, TD duplicated *ZmTLC* genes, as well as *ZmTLC10* could be important candidates for further functional analysis.

## 4. Materials and Methods

### 4.1. Data Sources and Identification of TLC Proteins

Gene models and proteomes of 31 plant genomes were downloaded and utilized in the present study. Genome and annotation data of maize v4.41 were downloaded from the MaizeGDB website [35]. Genome Annotation resources of *Ostreococcus lucimarinus*, *Micromonas pusilla*, *Volvox cartei*, *Chlamydomonas reinhardtii*, *Sphagnum fallax*, *Physcomitrella patens*, *Marchantia polymorpha*, *Selaginella moellendorffii*, *Amborella trichopoda*, *Spirodela polyrhiza*, *Musa acuminata*, *Ananas comosus*, *Sorghum bicolor*, *Brachypodium distachyon*, *Oryza sativa*, *S. lycopersicum*, *Capsicum annuum*, *Medicago truncatula*, *Phaseolus vulgaris*, *Glycine max*, *Prunus persica*, *Malus domestica*, *Populus trichocarpa*, *Eucalyptus grandis*, *Gossypium raimondii*, *Brassica rapa*, *Arabidopsis thaliana*, *Citrus maxima*, and *Citrus sinensis* were downloaded from Phytozome (http://phytozome.jgi.doe.gov/pz/portal.html). Annotation of the *Ginkgo biloba* genome was downloaded from previous literature [36] (Table S1). Genome annotation of *Mus musculus*, *Caenorhabditis elegans*, and *Saccharomyces cerevisiae* was downloaded from the ensembl genome [37] (https://asia.ensembl.org/index.html) (Table S1). A local perl script “Pfam_scan perl” downloaded from HMMER3.1 was used to search all surveyed proteomes against local Pfam library (http://hmmer.org/). All hits were subjected to the Pfam database, with the E-value set to default. Candidate TLC proteins were selected with the TRAM_LAG1_CLN8 domain. The longest transcript of each candidate gene was selected as representative for further analysis if primary transcripts were not provided [38].

### 4.2. Collinearity and Gene Duplication mOdes Prediction

MCScanX package was utilized to detect collinearity within or cross species genomes, as well as duplication modes in which angiosperms TLC proteins involved [19]. MCScanX implemented whole-genome BLASTP results to compute synteny/collinearity blocks within or among species. This package can also efficiently classify the gene duplication mechanism underlying one gene family evolution, focusing on WGD/SD and local (proximal and tandem) duplication based on copy number and their genomic distribution. To detect transposed duplication in maize, MCScanX-transposed was applied [39]. Four monocot species, including *O. sativa*, *S. italica*, *B. distachyon*, and *S. bicolor*, were employed to be outgroups. To deduce collinear communities between maize, *O*. *sativa*, *S*. *bicolor*, and *Arabidopsis*, Synteny method was utilized [21].

### 4.3. Calculation of Synonymous to Non-Synonymous Ratio

Synonymous to non-synonymous ratio of duplicated *TLC* gene pairs was calculated to estimate selection pressure. Their nucleotide’s CDS was first selected and translated to amino acid sequences and aligned by clustalw2. The codon-based maximum likelihood (CodeML) method in the PAML4.0 package was used to estimate the average non-synonymous/synonymous mutation ratio (Ka/Ks ratio) with a one-ratio model [40].

### 4.4. Phylogenetic Analysis

TLC proteins from all surveyed species were selected and aligned with MAFFT, using auto strategy [41]. TrimAl v1.2 was utilized to identify and delete gaps and obscure aligned sequences, with the parameter of -automated1 [42]. Then aligned sequences were further tested in ProtTest3.4 to select the best-fit amino acid substitution model for the ML phylogenetic tree construction [43]. The best model according to AIC was JTT + G + F (-lnL = 67552.85). Finally, the trimmed aligned protein sequences were submit to phyML 3.0 to construct the ML phylogenetic tree [44]. Fast approximate likelihood-based measures of branch supports (Shimodaira–Hasegawa approximate likelihood ratio test, SH-aLRT) were used for the branches. Other parameters were set based on the results of ProtTest test (amino acid frequencies = observed; gamma shape = 1.698). Additionally, another ML tree with only maize TLC proteins was also constructed with the same method, while the best model according to AIC was JTT + G + I + F (-lnL = 69201.52, gamma shape = 3.686; Proportion of invariable sites = 0.012). Obtained trees were edited with ITOL and MEGA 7.0 [45].

### 4.5. Motif Composition and Gene Structure Analysis

To detect conserved motifs in TLC proteins, a local MEME program was utilized with the command line as follows: meme protein_sequnece.fas -o result -protein -evt 0.05 -maxsize 10000000 -nmotifs 17 [46]. Gene structures of *ZmTLCs* were drawn in the GSDS (v2.0) website [47].

### 4.6. Calling of Single Nucleotide Polymorphisms (SNPs) and Calculation of Genetic Parameters

Re-sequencing data of 30 inbred and 22 wild maize were downloaded from peazea [22], with sequencing depth ≥ 10 (Table S2). All the reads of selected samples were mapped against the maize reference genome (B73 v3.0) by BWA using default parameters [48]. Picard-MarkDuplicates (http://broadinstitute.github.io/picard/) and GATK-IndelRealigner were used to correct mapping results. Finally, GATK-UnifiedGenotyper was utilized to detect SNPs in each maize individual at corresponding gene loci. Nucleotide sequences of *ZmTLC* genes in each individual maize population were obtained by local perl script (Supplementary File 2). If the non-determined nucleotide (N) exceeds 10%, the sequence would be eliminated in further analysis due to low coverage. Furthermore, nucleotide variation was estimated as the ratio between SNP numbers and corresponding CDS length. Pair-wise fixation index (Fst) and Tajima’D were calculated by Arlequin3.1 [49].

### 4.7. Expression Profiles of ZmTLCs

The expression profiles were downloaded from the MaizeGDB website and dataset source is Walley 2016 (Briggs Labs) [35,50]. RNA was obtained from 23 distinct tissues. All reads were mapped by the STAR 2.5.3 aligner [51] and FPKM abundances were calculated by Cufflinks 2.2.1 with default settings [52].

### 4.8. Planting and Cultivation of Corn

Four maize lines were germinated in sand for 2 days at 28 °C, and then grown in pots to 4th leaf stage maize seedlings at 25–30 °C, under a usual photoperiod. The second and third leaves of maize seedlings were obtained after they were wiped with 75% alcohol. All the samples were frozen immediately in liquid nitrogen and stored at −80 °C for the extraction of RNA.

### 4.9. Pathogen Cultivation and Infection

One fungus pathogen, *Curvularia lunata*, and one bacterial pathogen, *Pantoea stewartii* (Pnss), were utilized in the present study. The *Curvularia lunata* strain was cultured on potato dextrose agar (PDA) for 4–5 days at 28 °C under darkness [53]. Then the fungus dishes were transferred into corn kernels which were soaked for 2 days and sterilized for 1 h at 121 °C for 1 week at 28 °C under darkness. The corn kernels were sterilized with UV for 1 h after the hyphae of their surface was washed with sterile water and then moisturizing cultured for 2–3 days at room temperature. The conidia spore suspension was prepared by eluting the conidia growth on corn kernels and adding it into distilled water. The spore concentration was determined by hemocytometer. The fungal fluid in tubes (50 mL) was centrifuged at 4000 rpm for 15 min at 4 °C to collect the conidia pellets. The spore suspension of 10^6^ cfu/mL in 2% sucrose and 0.05% Tween-20 was sprayed on B73 and chang7-2, respectively, using air sprayers. At the same time, the project contained 2% sucrose and 0.02% Tween-20 which was used as a negative control. The leaves were sampled at 0, 12, 24, and 48 h after inoculation and three seedlings from each inbred line were obtained as samples.

The Pnss strain was streaked on nutrient agar (NA) for 3 days at 25 °C under darkness and a single colony was picked up and the culture was expanded for 48 h [54]. The bacteria was shifted into a liquid medium (50 mL) to oscillate at 25 °C (OD_540_ = 0.57). The bacterial fluid was centrifuged at 3000 rpm for 5 min at 4 °C to collect the bacterial cells. The bacterial suspension, which was 10^7–8^ cfu/mL in phosphate buffer solution (PBS) and 0.05% Tween-20, was injected into the stalks of T1 using syringes. The negative control was injected with phosphate buffer solution (PBS) and 0.05% Tween-20. The leaves were sampled at 0, 12, 24, and 48 h after inoculation and three seedlings from each inbred line were obtained as samples.

### 4.10. Inoculation Method of Fumonisin B1

For fumonisin B_1_ (FB_1_) treatment, 10 µM FB_1_ was prepared with Fumonisin B1 Standard (>96%) and sterile water. The liquid was irrigated around the root of every B73 inbred line seedling to 20 mL. The leaves were sampled at 0, 12, 24, and 48 h after irrigation and three seedlings from each inbred line were obtained as controls.

### 4.11. RNA Extraction and RT-qPCR

RNA was extracted from pathogen or toxins treated leaves and the quality was evaluated by electrophoresis strips and spectrophotometric measurement [55,56]. Then obtained RNA was reverse transcripted to complementary DNA (cDNA) using a HiScript®IIIRT SuperMix for qPCR (+gDNA wiper) Kit (Vazyme). In RT-qPCR, each reaction had a total volume of 20 µL, consistind of 2 µL diluted cDNA, 10 µL of AceQ qPCR SYBR Green Master Mix (Vazyme), 1 µL forward and reverse primers, and 6 µL RNA-free water. Three technical replications were performed per sample. Cycling of qPCR validation was 95 °C 5 min, followed with 40 cycles of 95 °C for 10 s, 60 °C for 30 s. *ZmActin1* (Zm00001d010159) and *ZmGADPH* (Zm00001d049641) were used as internal controls, and primers were designed with Primer Premier Software (v 5.0) [57] (Table S3). Primers were selected according to the following criteria: oligo length should be between 15 and 25 bases; melting temperature (Tm) should between 55 °C and 65 °C; no hairpins and heterodimers should exist between two primers. A BLAST alignment was performed in the MaizeGDB website to ensure the specificity of selected primers [35]. All available primers were listed in Table S3. The relative expression levels of these genes were calculated by the Delta-Delta Ct method with the geometric mean of the two reference genes [58]. Finally, results were displayed by Origin2018.

## Figures and Tables

**Table 1:** Information of maize TLC proteins.

Gene Name	v4 Gene ID	Chromosomal Location	Amino Acid Length	Transcript Number	PI ^a^	MW/Da ^b^	Duplication Type ^c^
*ZmTLC1*	Zm00001d028534	Chr1:38449782-38457129	282	9	7.61	32894.85	TD/WD
*ZmTLC2*	Zm00001d030772	Chr1:159066629:159067242	189	1	9.96	21106.89	TD
*ZmTLC3*	Zm00001d034572	Chr1:296603347-296604114	255	1	9.32	28436.61	WD
*ZmTLC4*	Zm00001d034948	Chr1:306476631-306490356	269	10	9.12	30978.78	/
*ZmTLC5*	Zm00001d005671	Chr2:182856428-182859197	313	3	7.06	36695.95	TD/WD
*ZmTLC6*	Zm00001d042699	Chr3:177342649-177348724	278	2	9.3	31148.42	WD
*ZmTLC7*	Zm00001d043088	Chr3:188898108-188902810	263	4	9.06	30130.48	/
*ZmTLC8*	Zm00001d050910	Chr4:130225510-130230340	331	4	8.9	39114.39	WD
*ZmTLC9*	Zm00001d013028	Chr5:3464279-3465049	256	1	9.17	28548.65	WD/TD
*ZmTLC10*	Zm00001d013582	Chr5:14847771-14851912	267	8	9.29	30711.55	/
*ZmTLC11*	Zm00001d016993	Chr5:182188749-182194083	327	16	9.42	38409.8	WD
*ZmTLC12*	Zm00001d017912	Chr5:210036826-210039426	307	1	6.46	36281.25	/
*ZmTLC13*	Zm00001d020296	Chr7:104757541-104760661	313	2	6.08	36529.66	WD
*ZmTLC14*	Zm00001d008305	Chr8:4713419-4715161	250	2	8.62	26785.56	/
*ZmTLC15*	Zm00001d010723	Chr8:125888822-125894023	280	1	8.5	31996.93	/
*ZmTLC16*	Zm00001d012265	Chr8:171473506-171483308	278	2	9.18	31161.52	WD
*ZmTLC17*	Zm00001d012659	Chr8:178077109-178082439	275	9	9.15	31336.05	/
*ZmTLC18*	Zm00001d047854	Chr9:143512868-143522027	281	7	8.18	32704.65	WD

^a^: Isoelectronic point. ^b^: Molecular weight. ^c^: TD represents transposed duplication; WD represents whole-genome/segmental duplication.

**Table 2:** Population genetics analysis of *ZmTLCs* within/between inbred and wild maize populations.

Gene Symbol	Pi ^1^		Tajima’D		Fst
	Inbred	Wild	Inbred	Wild	
*ZmTLC1*	0.000	0.000	0	−1.16467	0
*ZmTLC4*	0.001	0.002	−0.99368	−0.77806	0.54606 **
*ZmTLC5*	0.004	0.004	−1.13153	−1.06658	0.0111
*ZmTLC6*	0.001	0.004	−0.24788	−0.33106	0.16072 **
*ZmTLC7*	0.008	0.007	0.69553	−1.04302	0.04434
*ZmTLC8*	0.004	0.004	2.59687	−0.79777	0.18031 *
*ZmTLC9*	-	0.007	-	−0.1566	−0.19835
*ZmTLC10*	0.000	0.003	**−1.73263	−0.82916	0.13773 **
*ZmTLC12*	0.004	0.004	−0.19355	−0.66553	0.06993
*ZmTLC13*	0.001	0.001	0.25259	−0.02381	0.06414
*ZmTLC14*	-	0.007	0	0.60901	−0.44444
*ZmTLC16*	0.005	0.005	−0.05722	−0.42687	0.15669
*ZmTLC17*	0.002	0.005	0.66439	−0.74782	0.01362
*ZmTLC18*	0.001	0.001	1.63299	*−1.45138	−0.12821

^1^: Pi means nucleotide diversity. *: significant; **: highly significant.

## Supplementary Materials

The following are available online at https://www.mdpi.com/1422-0067/20/21/5484/s1, **Supplementary File 1:** Figure S1. Correlation analysis between TLC numbers with genome-wide protein-encoding gene numbers (A), genome size (B) and chromosome number (C). Figure S2. The ML phylogenetic tree built by TLC proteins from 31 plant species and 3 outgroup species. Table S1. Genome information of 31 plant species and 3 outgroup species. Table S2. Inbred and wild maize used in the present study. Table S3. Primer sequences used for RT-qPCR analysis. Table S4. Ka, Ks, and Ka/Ks ratio of WGD/SD and LD duplicated gene pairs in surveyed angiosperms. **Supplementary File 2:** Coding sequences of *ZmTLCs* assembled from inbred and wild resequencing data.
