# Supplementary material for: Whole-Genome and Transposed Duplication Contributes to the Expansion and Diversification of TLC Genes in Maize
Source: Int J Mol Sci. 2019 Nov 4;20(21):5484. doi: 10.3390/ijms20215484 (PMC6862079; doi:10.3390/ijms20215484)
Supplement: Supplementary file 1 [file ijms-20-05484-s001.zip › ijms-598091-supplementary/Supplementary file1.pdf]

FigureS1 Correlation analysis between TLC numbers with genome-wide protein-encoding gene numbers (A), genome size (B) and chromosome number (C), respectively.

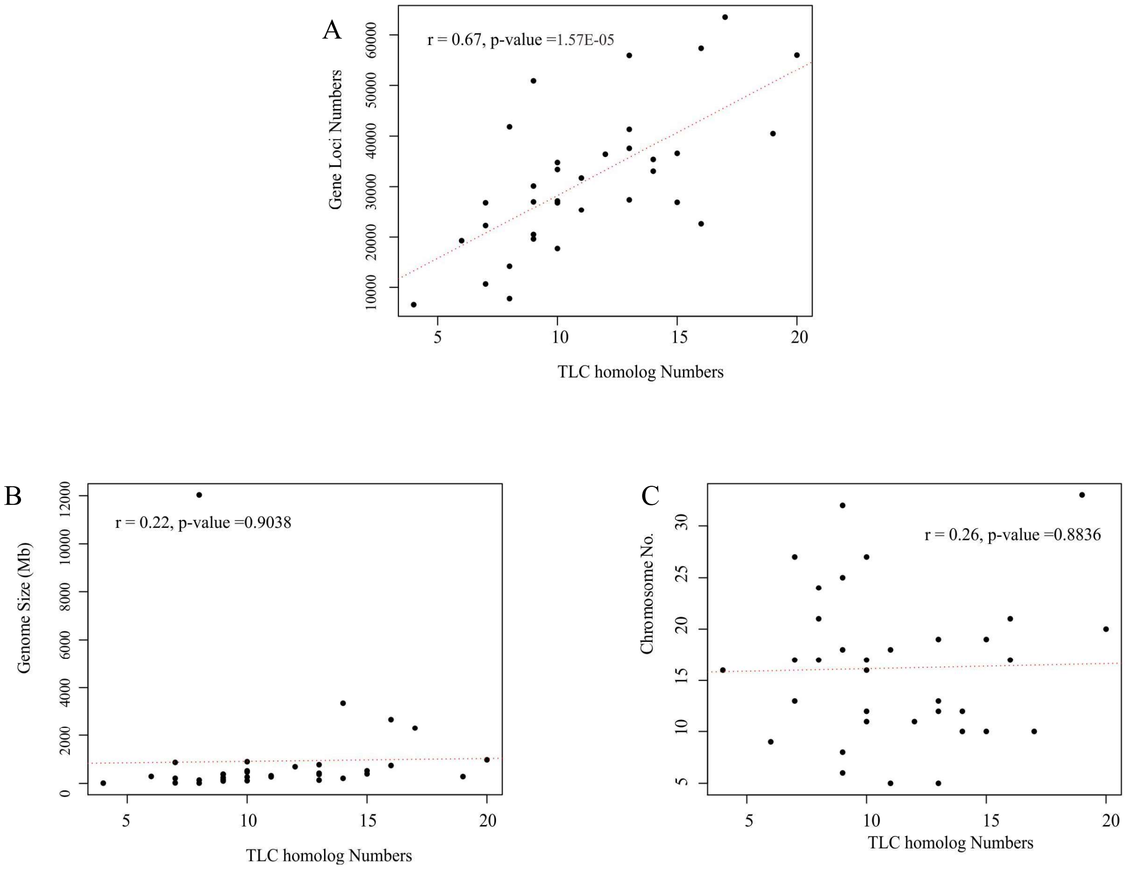

FigureS2 The ML Phylogenetic tree built by TLC proteins from 31 plant species and three out-group species.

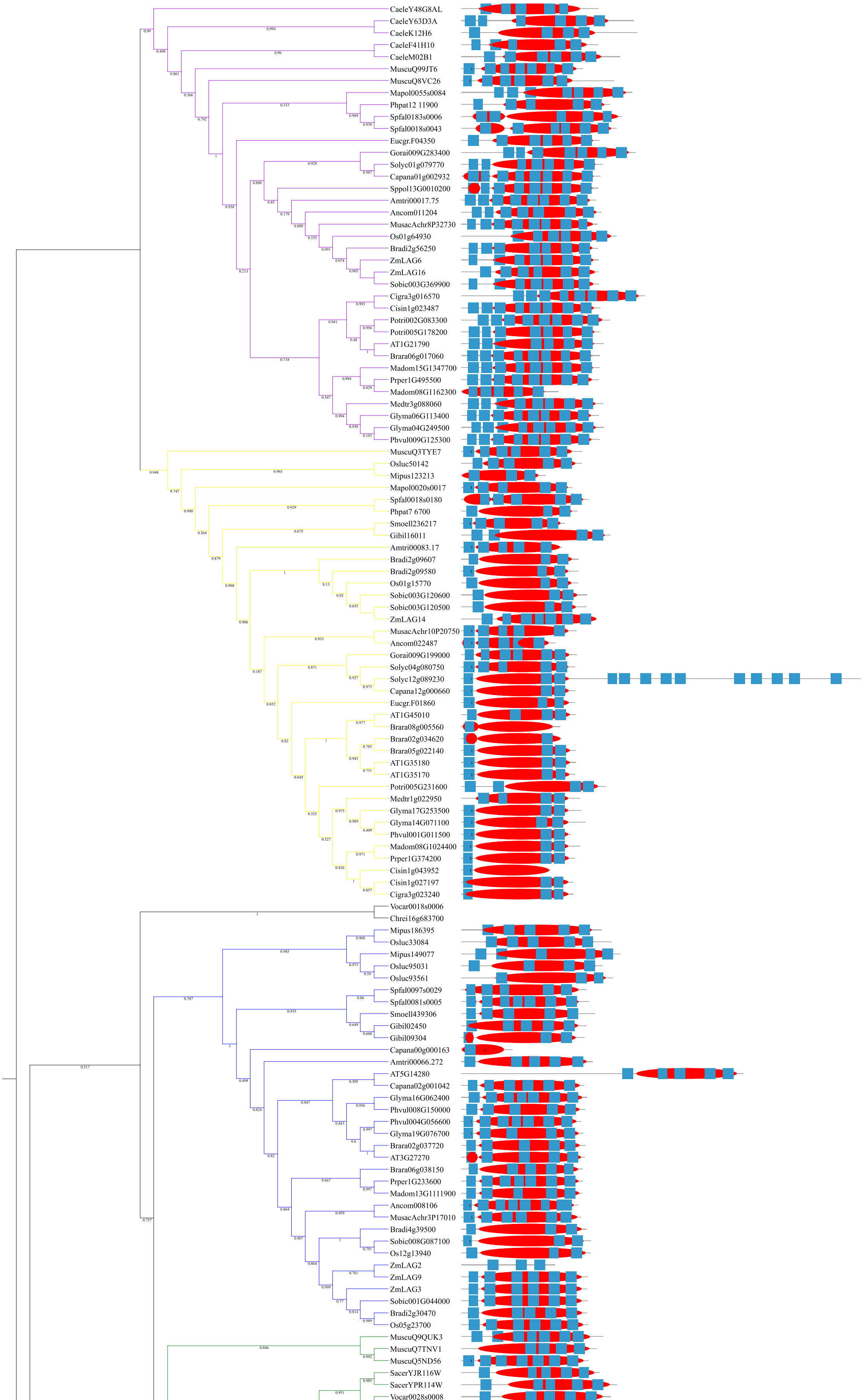
CaeleY48G8AL
CaeleY63D3A
CaeleK12H6
CaeleF41H10
CaeleM02B1
MuscuQ99JT6
MuscuQ8VC26
Mapol0055s0084
Phpat12 11900
Spfal0183s0006
Spfal0018s0043
Eucgr.F04350
Gorai009G283400
Solyc01g079770
Capana01g002932
Sppol13G0010200
Amtri00017.75
Ancom011204
MusacAchr8P32730
Os01g64930
Bradi2g56250
ZmLAG6
ZmLAG16
Sobic003G369900
Cigra3g016570
Cisin1g023487
Potri002G083300
Potri005G178200
AT1G21790
Brara06g017060
Madom15G1347700
Prper1G495500
Madom08G1162300
Medtr3g088060
Glyma06G113400
Glyma04G249500
Phvul009G125300
MuscuQ3TYE7
Osluc50142
Mipus123213
Mapol0020s0017
Spfal0018s0180
Phpat7 6700
Smoell236217
Gibil16011
Amtri00083.17
Bradi2g09607
Bradi2g09580
Os01g15770
Sobic003G120600
Sobic003G120500
ZmLAG14
MusacAchr10P20750
Ancom022487
Gorai009G199000
Solyc04g080750
Solyc12g089230
Capana12g000660
Eucgr.F01860
AT1G45010
Brara08g005560
Brara02g034620
Brara05g022140
AT1G35180
AT1G35170
Potri005G231600
Medtr1g022950
Glyma17G253500
Glyma14G071100
Phvul001G011500
Madom08G1024400
Prper1G374200
Cisin1g043952
Cisin1g027197
Cigra3g023240
Vocar0018s0006
Chrei16g683700
Mipus186395
Osluc33084
Mipus149077
Osluc95031
Osluc93561
Spfal0097s0029
Spfal0081s0005
Smoell439306
Gibil02450
Gibil09304
Capana00g000163
Amtri00066.272
AT5G14280
Capana02g001042
Glyma16G062400
Phvul008G150000
Phvul004G056600
Glyma19G076700
Brara02g037720
AT3G27270
Brara06g038150
Prper1G233600
Madom13G1111900
Ancom008106
MusacAchr3P17010
Bradi4g39500
Sobic008G087100
Os12g13940
ZmLAG2
ZmLAG9
ZmLAG3
Sobic001G044000
Bradi2g30470
Os05g23700
MuscuQ9QUK3
MuscuQ7TNV1
MuscuQ5ND56
SacerYJR116W
SacerYPR114W
Vocar0028s0008

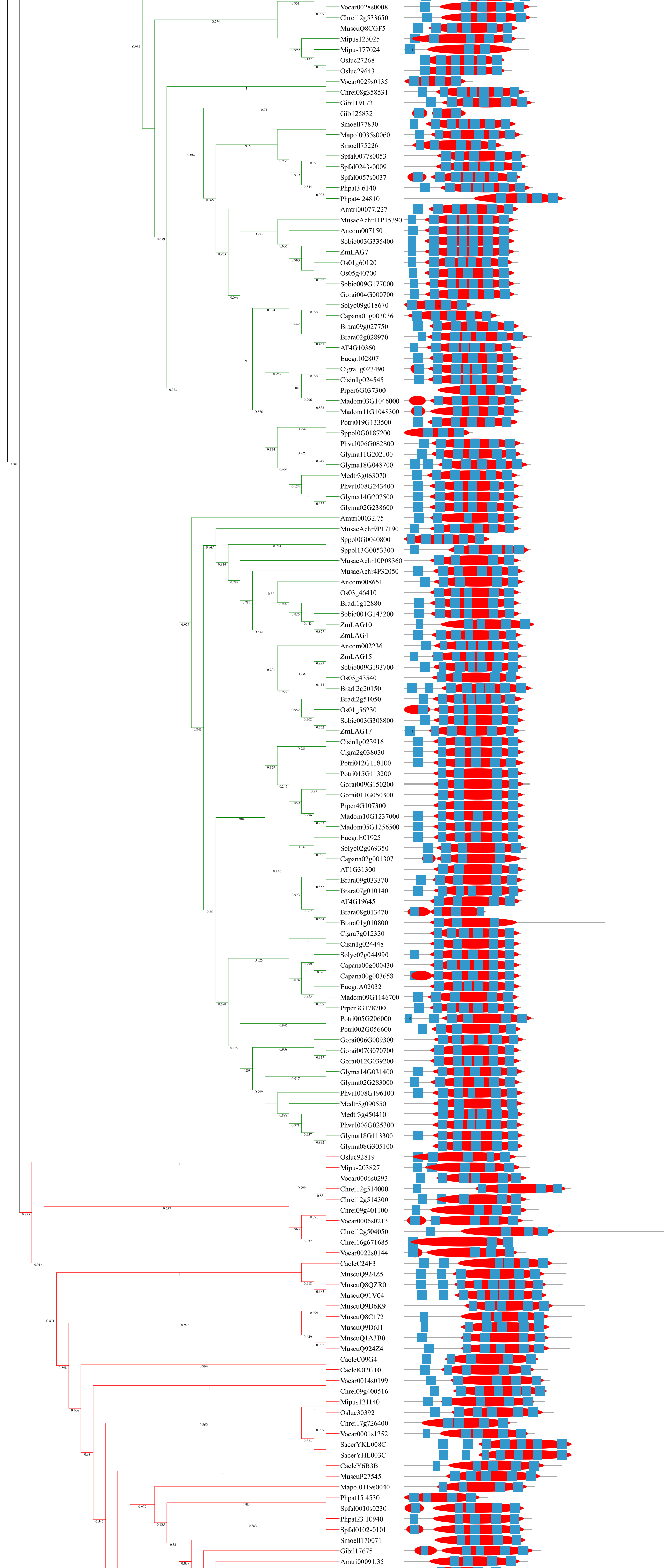
Vocar0028s0008
Chrei12g533650
MuscuQ8CGF5
Mipus123025
Mipus177024
Osluc27268
Osluc29643
Vocar0029s0135
Chrei08g358531
Gibil19173
Gibil25832
Smoell77830
Mapol0035s0060
Smoell75226
Spfal0077s0053
Spfal0243s0009
Spfal0057s0037
Phpat3 6140
Phpat4 24810
Amtri00077.227
MusacAchr11P15390
Ancom007150
Sobic003G335400
ZmLAG7
Os01g60120
Os05g40700
Sobic009G177000
Gorai004G000700
Solyc09g018670
Capana01g003036
Brara09g027750
Brara02g028970
AT4G10360
Eucgr.I02807
Cigra1g023490
Cisin1g024545
Prper6G037300
Madom03G1046000
Madom11G1048300
Potri019G133500
Sppol0G0187200
Phvul006G082800
Glyma11G202100
Glyma18G048700
Medtr3g063070
Phvul008G243400
Glyma14G207500
Glyma02G238600
Amtri00032.75
MusacAchr9P17190
Sppol0G0040800
Sppol13G0053300
MusacAchr10P08360
MusacAchr4P32050
Ancom008651
Os03g46410
Bradi1g12880
Sobic001G143200
ZmLAG10
ZmLAG4
Ancom002236
ZmLAG15
Sobic009G193700
Os05g43540
Bradi2g20150
Bradi2g51050
Os01g56230
Sobic003G308800
ZmLAG17
Cisin1g023916
Cigra2g038030
Potri012G118100
Potri015G113200
Gorai009G150200
Gorai011G050300
Prper4G107300
Madom10G1237000
Madom05G1256500
Eucgr.E01925
Solyc02g069350
Capana02g001307
AT1G31300
Brara09g033370
Brara07g010140
AT4G19645
Brara08g013470
Brara01g010800
Cigra7g012330
Cisin1g024448
Solyc07g044990
Capana00g000430
Capana00g003658
Eucgr.A02032
Madom09G1146700
Prper3G178700
Potri005G206000
Potri002G056600
Gorai006G009300
Gorai007G070700
Gorai012G039200
Glyma14G031400
Glyma02G283000
Phvul008G196100
Medtr5g090550
Medtr3g450410
Phvul006G025300
Glyma18G113300
Glyma08G305100
Osluc92819
Mipus203827
Vocar0006s0293
Chrei12g514000
Chrei12g514300
Chrei09g401100
Vocar0006s0213
Chrei12g504050
Chrei16g671685
Vocar0022s0144
CaeleC24F3
MuscuQ924Z5
MuscuQ8QZR0
MuscuQ91V04
MuscuQ9D6K9
MuscuQ8C172
MuscuQ9D6J1
MuscuQ1A3B0
MuscuQ924Z4
CaeleC09G4
CaeleK02G10
Vocar0014s0199
Chrei09g400516
Mipus121140
Osluc30392
Chrei17g726400
Vocar0001s1352
SacerYKL008C
SacerYHL003C
CaeleY6B3B
MuscuP27545
Mapol0119s0040
Phpat15 4530
Spfal0010s0230
Phpat23 10940
Spfal0102s0101
Smoell170071
Gibil17675
Amtri00091.35

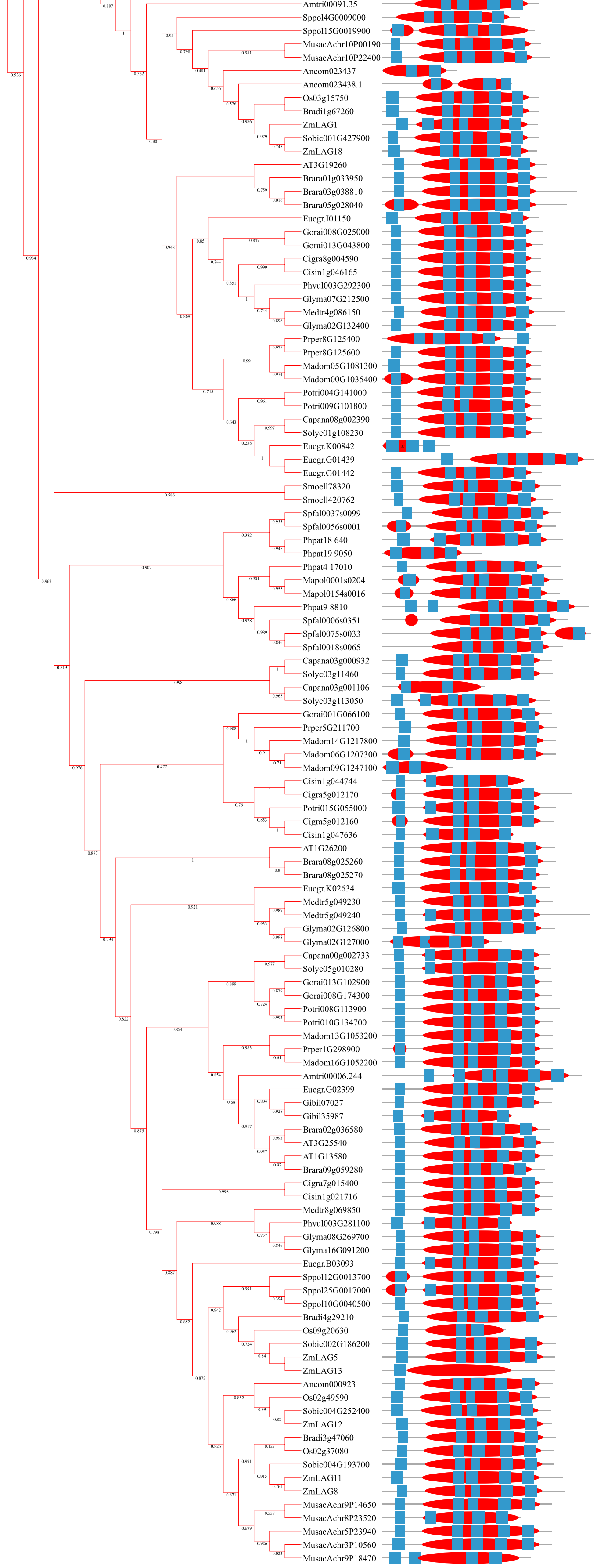
Amtri00091.35
Sppol4G0009000
Sppol15G0019900
MusacAchr10P00190
MusacAchr10P22400
Ancom023437
Ancom023438.1
Os03g15750
Bradi1g67260
ZmLAG1
Sobic001G427900
ZmLAG18
AT3G19260
Brara01g033950
Brara03g038810
Brara05g028040
Eucgr.I01150
Gorai008G025000
Gorai013G043800
Cigra8g004590
Cisin1g046165
Phvul003G292300
Glyma07G212500
Medtr4g086150
Glyma02G132400
Prper8G125400
Prper8G125600
Madom05G1081300
Madom00G1035400
Potri004G141000
Potri009G101800
Capana08g002390
Solyc01g108230
Eucgr.K00842
Eucgr.G01439
Eucgr.G01442
Smoell78320
Smoell420762
Spfal0037s0099
Spfal0056s0001
Phpat18 640
Phpat19 9050
Phpat4 17010
Mapol0001s0204
Mapol0154s0016
Phpat9 8810
Spfal0006s0351
Spfal0075s0033
Spfal0018s0065
Capana03g000932
Solyc03g11460
Capana03g001106
Solyc03g113050
Gorai001G066100
Prper5G211700
Madom14G1217800
Madom06G1207300
Madom09G1247100
Cisin1g044744
Cigra5g012170
Potri015G055000
Cigra5g012160
Cisin1g047636
AT1G26200
Brara08g025260
Brara08g025270
Eucgr.K02634
Medtr5g049230
Medtr5g049240
Glyma02G126800
Glyma02G127000
Capana00g002733
Solyc05g010280
Gorai013G102900
Gorai008G174300
Potri008G113900
Potri010G134700
Madom13G1053200
Prper1G298900
Madom16G1052200
Amtri00006.244
Eucgr.G02399
Gibil07027
Gibil35987
Brara02g036580
AT3G25540
AT1G13580
Brara09g059280
Cigra7g015400
Cisin1g021716
Medtr8g069850
Phvul003G281100
Glyma08G269700
Glyma16G091200
Eucgr.B03093
Sppol12G0013700
Sppol25G0017000
Sppol10G0040500
Bradi4g29210
Os09g20630
Sobic002G186200
ZmLAG5
ZmLAG13
Ancom000923
Os02g49590
Sobic004G252400
ZmLAG12
Bradi3g47060
Os02g37080
Sobic004G193700
ZmLAG11
ZmLAG8
MusacAchr9P14650
MusacAchr8P23520
MusacAchr5P23940
MusacAchr3P10560
MusacAchr9P18470

TableS1 Genome information of 31 plant species and three outgroup species.

Taxonomy	Species name	Abrr.	Genome Size(Mb)	Gene Loci number	Chromsome number	TLC number
Bathycoccaceae	*Ostreococcus lucimarinus*	Osluc	13.2	7796	21	8
Mamiellophyceae	*Micromonas pusilla*	Mipus	22	10660	17	7
Chlamydomonadaceae	*Chlamydomonas reinhardtii*	Chrei	111.1	17741	17	10
Chlorophyceae	*Volvox carteri*	Vocar	138	14247	17	8
Bryophyta	*Physcomitrella patens*	Phpat	473	33362	27	10
Sphagnaceae	*Sphagnum fallax*	Spfal	395	26939	19	15
Marchantiaceae	*Marchantia polymorpha*	Mapol	290	19287	9	6
Selaginellaceae	*Selaginella_moellendorffii*	Smoell	212.5	22273	27	7
Ginkgoaceae	*Ginkgo biloba*	Gibil	12032	41840	24	8
Amborellaceae	*Amborella trichopoda*	Amtri	870	26846	13	7
Musaceae	*Musa acuminata*	Musac	523	36528	10	15
Bromeliaceae	*Ananas comosus*	Ancom	382	27024	25	9
Araceae	*Spirodela polyrhiza*	Sppol	158.00	19623	32	9
Poaceae	*Zea mays*	Zm	2300	63480	10	18
Poaceae	*Sorghum Bicolor*	Sobic	211	33032	10	14
Poaceae	*Brachypodium distachyon*	Bradi	272	31694	5	11
Poaceae	*Oryza sativa*	Os	372	55986	12	13
Solanaceae	*Capsicum annuum*	Capana	3349	35336	12	14
Solanaceae	*Solanum lycopersicum*	Solyc	900	34727	12	10
Salicaceae	*Populus trichocarpa*	Potri	422.9	41335	19	13
Rosaceae	*Prunus persica*	Prper	265	26837	16	10
Rosaceae	*Malus domestica*	Madom	742.3	57386	17	16
Fabaceae	*Medicago truncatula*	Medtr	241	50894	8	9
Fabaceae	*Phaseolus vulgaris*	Phvul	521.1	27197	11	10
Fabaceae	*Glycine max*	Glyma	978	56044	20	20
Myrtaceae	*Eucalyptus grandis*	Eucgr	691	36349	11	12
Malvaceae	*Gossypium raimondii*	Gorai	775	37505	13	13
Rutaceae	*Citrus grandis*	Cigra	380	30120	18	9
Rutaceae	*Citrus sinensis*	Cisin	319	25376	18	11
Brassicaceae	*Brassica rapa*	Brara	283.8	40492	33	19
Brassicaceae	*Arabidopsis thaliana*	AT	135	27416	5	13
nematode	*Caenorhabditis elegans*	Caele	100	20512	6	9
Ascomycota	*Saccharomyces cerevisiae*	Sacer	12	6604	16	4
mammalia	*Mus musculus*	Muscu	2662.4	22619	21	16

TableS2 Inbred and wild maize used in the present study.

Accession number	Name	Type
1	B73_SRR1575495	Inbred
2	CH10_SRR1575496	Inbred
3	D06_SRR1575497	Inbred
4	D09_SRR1575498	Inbred
5	D152_SRR1575499	Inbred
6	DK105_SRR1575500	Inbred
7	EC169_SRR1575501	Inbred
8	EC49A_SRR1575502	Inbred
9	EP1_SRR1575503	Inbred
10	EP44_SRR1575504	Inbred
11	EZ5_SRR1575505	Inbred
12	F03802_SRR1575506	Inbred
13	F252_SRR1575508	Inbred
14	F283_SRR1575509	Inbred
15	F2_SRR1575507	Inbred
16	F353_SRR1575510	Inbred
17	F618_SRR1575511	Inbred
18	F64_SRR1575512	Inbred
19	F7_SRR1575513	Inbred
20	F98902_SRR1575514	Inbred
21	FF0721H-7_SRR1575515	Inbred
22	Lo11_SRR1575516	Inbred
23	Mo17_SRR1575517	Inbred
24	PH207_SRR1575518	Inbred
25	UH006_SRR1575520	Inbred
26	UH007_SRR1575521	Inbred
27	UH009_SRR1575522	Inbred
28	UH250_SRR1575523	Inbred
29	UH304_SRR1575524	Inbred
30	W117_SRR1575525	Inbred
31	TIL01	Wild
32	TIL02	Wild
33	TIL03	Wild
34	TIL04-TIP285	Wild
35	TIL04-TIP454	Wild
36	TIL05	Wild
37	TIL06-TIP260	Wild
38	TIL06-TIP496	Wild
39	TIL07	Wild
40	TIL08	Wild
41	TIL09	Wild

42	TIL10	Wild
43	TIL11	Wild
44	TIL12	Wild
45	TIL14-TIP498	Wild
46	TIL15	Wild
47	TIL16	Wild
48	TIL17	Wild
49	TIL25-TIP489	Wild
50	TIP489.2	Wild
51	TIP498.2	Wild
52	Teosinte	Wild

TableS3 Primer sequences used for RT-qPCR analysis.

Gene name	Forward primer (5'-3')	Reverse primer (5'-3')
ZmTLC1	CTTCTGTTCAAGCATGGGTCCTCA	TTTCCCAAGCAACAAGAGCACC
ZmTLC2	CTTCGGTGTCTCCACCACAATC	CAGCAGGTCAGGAAGACGAAGA
ZmTLC3	GGTGGCTGTTCCCGTTGTTC	GATCAGGCAGCTGGCGC
ZmTLC4	AGATGGCGACGATGACTGC	AGCACAGAGGTGTACGGAACGA
ZmTLC5	TCCTAGAGATTGGGAAGATGGC	GGAACATAACGAGCCGAAGAAGTA
ZmTLC6	CGCCGTATCGCTTCTCCAC	ACTGCCACCAAATAGTTCTTCATG
ZmTLC7	TCTTCACCGAAATCACGACTCC	ATTATCCGAGCAATCAGCCATC
ZmTLC8	TGCTACTGTCGTCCTGATTGTT	CGGAGAAGAACCCATGAAATAAC
ZmTLC9	CGTCATTCCGTGGTGGGTG	CTTTCTTTGACTTGGGAGGGG
ZmTLC10	GCCTTGATGAGGGACTATCTGCT	CAATATTCGAGTGAAATCGTAAGCC
ZmTLC11	GGTGGGTTTTAATATATCGGATGC	TGAATCTCGTGAGGAACAATGC
ZmTLC12	GGTTCAGTTGTCCTTGCCATTCAT	GCTCGTGCTCCAGAGAATCCAG
ZmTLC13	CGTGGCATCGTGGATACTTCTC	TTTCTCCTTGTCCAGGGTCAGC
ZmTLC14	GCGTGCTGCCACCTCAAC	CTTGAGCATCTCCCGCAGGT
ZmTLC15	CAATGTTTGTTACTTGGCTGATGG	TAGTGAACAGTATGGTGGGGGC
ZmTLC16	CTCGTCGCTGATGCTCACAAAT	CAATCCTGATGGCTCTGCTGGT
ZmTLC17	GTCCTTCACCATATGCTGTCGC	ATCGGAGGTTGATTCCAGGTGT
ZmTLC18	CGCCTCGTCTACAAGCCATTA	CTTGACAATCTTTGCTTGCCTG
ZmGADPH	CTTCGGCATTGTTGAGGGTTTG	TCCTTGGCTGAGGGTCCGTC
ZmActin1	GGGATTGCCGATCGTATGAG	GAGCCACCGATCCAGACACT

TableS4 Ka, Ks and Ka/Ks ratio of WGD/SD and LD duplicated gene pairs in surveyed angiosperms.

Gene1	Gene2	Ks	Ka	Ka/Ks	Duplication pairs
Aco023437	Aco023438	9.1895	1.5284	0.1663	Aco_LD1
Aco002236	Aco008651	0.922	0.1188	0.1288	Aco_WGD1
AT1G35170.1	AT1G35180.1	0.0572	0.0292	0.5112	AT_LD1
AT1G31300.1	AT4G19645.1	3.4969	0.1849	0.0529	AT_WGD1
Bradi2g09580	Bradi2g09607	0.8721	0.135	0.1548	Bd_LD1
Bradi2g20150	Bradi2g51050	0.8881	0.1725	0.1943	Bd_WGD1
BraA08g025260.3C	BraA08g025270.3C	0.1145	0.0737	0.6431	Brapa_LD1
BraA01g010800.3C	BraA08g013470.3C	0.2289	0.0884	0.3864	Brapa_WGD1
BraA01g010800.3C	BraA09g033370.3C	2.8028	0.2725	0.0972	Brapa_WGD2
BraA01g033950.3C	BraA03g038810.3C	54.0486	1.3985	0.0259	Brapa_WGD3
BraA01g033950.3C	BraA05g028040.3C	0.3769	0.1013	0.2687	Brapa_WGD4
BraA02g028970.3C	BraA09g027750.3C	0.2529	0.0647	0.256	Brapa_WGD5
BraA03g038810.3C	BraA05g028040.3C	51.9784	1.1822	0.0227	Brapa_WGD6
BraA07g010140.3C	BraA09g033370.3C	0.3335	0.0165	0.0495	Brapa_WGD7
BraA08g025260.3C	BraA09g059280.3C	3.5269	0.3445	0.0977	Brapa_WGD8
Cg5g012160.1	Cg5g012170.1	0.5174	0.1647	0.3183	Cgrandis_LD1
Cg5g012160.1	Cg7g015400.1	2.8258	0.2427	0.0859	Cgrandis_WGD1
Eucgr.G01439	Eucgr.G01442	0.0201	0.0205	1.019	Eu_LD1
Eucgr.B03093	Eucgr.G02399	0.9497	0.1327	0.1397	Eu_WGD1
Eucgr.G01439	Eucgr.I01150	1.5345	0.1925	0.1254	Eu_WGD2
Glyma.02G126800	Glyma.02G127000	0.0464	0.0135	0.2901	Gmax_LD1
Glyma.02G132400	Glyma.07G212500	0.0927	0.0285	0.307	Gmax_WGD1
Glyma.02G238600	Glyma.11G202100	0.4503	0.1471	0.3267	Gmax_WGD2
Glyma.02G238600	Glyma.14G207500	0.1312	0.0322	0.2451	Gmax_WGD3
Glyma.02G238600	Glyma.18G048700	0.451	0.1396	0.3096	Gmax_WGD4
Glyma.02G283000	Glyma.08G305100	0.3628	0.1078	0.2971	Gmax_WGD5
Glyma.02G283000	Glyma.14G031400	0.0894	0.0178	0.1985	Gmax_WGD6
Glyma.02G283000	Glyma.18G113300	0.3653	0.1088	0.298	Gmax_WGD7
Glyma.04G249500	Glyma.06G113400	0.1661	0.0461	0.2774	Gmax_WGD8
Glyma.08G305100	Glyma.14G031400	0.3776	0.0949	0.2513	Gmax_WGD9
Glyma.08G305100	Glyma.18G113300	0.0686	0.0251	0.3656	Gmax_WGD10
Glyma.11G202100	Glyma.14G207500	0.5063	0.1704	0.3366	Gmax_WGD11
Glyma.11G202100	Glyma.18G048700	0.0483	0.0276	0.572	Gmax_WGD12
Glyma.14G031400	Glyma.18G113300	0.3567	0.0922	0.2585	Gmax_WGD13
Glyma.14G071100	Glyma.17G253500	0.2205	0.056	0.2541	Gmax_WGD14
Glyma.14G207500	Glyma.18G048700	0.4909	0.1627	0.3315	Gmax_WGD15

Glyma.16G062400	Glyma.19G076700	2.0046	0.3119	0.1556	Gmax_WGD16
Gorai.008G025000.1	Gorai.013G043800.1	0.3998	0.1041	0.2603	Graim_WGD1
Gorai.008G174300.1	Gorai.013G102900.1	0.3881	0.0934	0.2406	Graim_WGD2
Gorai.009G150200.1	Gorai.011G050300.1	0.3756	0.1029	0.2738	Graim_WGD3
GSMUA_Achr3G10560_001	GSMUA_Achr5G23940_001	0.355	0.059	0.1661	Mac_WGD1
GSMUA_Achr3G10560_001	GSMUA_Achr9G18470_001	0.7028	0.3837	0.546	Mac_WGD2
GSMUA_Achr8G23520_001	GSMUA_Achr9G14650_001	0.3336	0.1335	0.4003	Mac_WGD3
Medtr5g049230.1	Medtr5g049240.1	0.3525	0.0948	0.2689	Mt_LD1
Medtr3g450410.1	Medtr5g090550.1	0.6917	0.0919	0.1329	Mt_WGD1
Os01g56230	Os05g43540	0.6953	0.1588	0.2284	Os_WGD1
Os01g60120	Os05g40700	0.8186	0.1601	0.1956	Os_WGD2
Potri.002G056600.1	Potri.005G206000.1	0.1912	0.0634	0.3316	Potri_WGD1
Potri.002G083300.1	Potri.005G178200.1	0.2665	0.0668	0.2507	Potri_WGD2
Potri.004G141000.1	Potri.009G101800.1	0.169	0.0689	0.408	Potri_WGD3
Potri.008G113900.1	Potri.010G134700.1	0.1884	0.0584	0.31	Potri_WGD4
Potri.012G118100.1	Potri.015G113200.1	0.1395	0.0379	0.2719	Potri_WGD5
Pp3c18_640	Pp3c19_9050	1.6134	0.3856	0.239	Ppaten_WGD1
Prupe.1G298900.1	Prupe.5G211700.1	1.1559	0.2387	0.2065	Ppersica_WGD1
Prupe.1G233600.1	Prupe.3G301200.1	4.4423	0.2962	0.0667	Ppersica_WGD2
Prupe.8G125400.1	Prupe.8G125600.1	0.1591	0.0994	0.6246	Ppersics_LD1
Sobic.003G120500	Sobic.003G120600	0.2886	0.0385	0.1335	Sbi_LD1
Sobic.003G308800	Sobic.009G193700	0.8609	0.1675	0.1945	Sbi_WGD1
Sobic.003G335400	Sobic.009G177000	1.1523	0.178	0.1545	Sbi_WGD2
Solyc04g080750.2.1	Solyc12g089230.1.1	0.863	0.1539	0.1783	Sly_WGD1
Spipo10G0040500	Spipo12G0013700	0.83	0.156	0.188	Spipo_WGD1
Spipo10G0040500	Spipo25G0017000	1.1894	0.184	0.1547	Spipo_WGD2
Spipo12G0013700	Spipo25G0017000	1.3714	0.1681	0.1225	Spipo_WGD3
Spipo15G0019900	Spipo4G0009000	1.3817	0.3589	0.2598	Spipo_WGD4
Zm00001d005671	Zm00001d020296	0.0886	0.0259	0.2922	Zm_WGD1
Zm00001d034572	Zm00001d013028	0.3092	0.0106	0.0343	Zm_WGD2
Zm00001d042699	Zm00001d012265	0.1321	0.0192	0.1456	Zm_WGD3
Zm00001d050910	Zm00001d016993	0.1617	0.0676	0.4179	Zm_WGD4
Zm00001d013028	Zm00001d030772	0.6296	0.1617	0.2569	Zm_TD1
Zm00001d005671	Zm00001d028534	3.072	0.5971	0.1944	Zm_TD2
